# Lifetime predictors of stroke in subjects without a diagnosis of hypertension: the aerobics center longitudinal study

**DOI:** 10.2147/NDT.S193842

**Published:** 2019-04-08

**Authors:** Iván Cavero-Redondo, Xuemei Sui, Steven N Blair, Carl J Lavie, Celia Álvarez-Bueno, Vicente Martínez-Vizcaíno

**Affiliations:** 1 Universidad de Castilla-La Mancha, Health and Social Research Center, Cuenca, Spain, celia.alvarezbueno@uclm.es; 2 Department of Exercise Science, Arnold School of Public Health, University of South Carolina, Columbia, SC, USA; 3 Department of Cardiovascular Diseases, John Ochsner Heart and Vascular Institute, Ochsner Clinical School, The University of Queensland School of Medicine, New Orleans, LA, USA; 4 Universidad Autónoma de Chile, Facultad de Ciencias de la Salud, Talca, Chile

**Keywords:** stroke, risk factors, cohort study, incidence, cerebrovascular disease

## Abstract

**Background and purpose:**

Although several studies have assessed the importance of traditional risk factors in predicting stroke, none have concurrently addressed the stroke-predicting ability of these risk factors across the lifespan of subjects without a hypertension (HTN) diagnosis. Thus, this study aimed to assess the importance of blood-pressure-related risk indicators, cardiorespiratory fitness (CRF), weight status, diabetes mellitus (DM), and lifestyle factors as predictors of stroke in different stages of life among non-hypertensive subjects.

**Materials and methods:**

This study was a long-term follow-up study including 33,254 men and 10,598 women from the Aerobics Center Longitudinal Study (ACLS) who were 18–100 years old and did not have a HTN diagnosis at baseline. Logistic regression models were constructed using forward selection procedures for each age category, with stroke occurrence as the dependent variable, and pulse pressure (PP), mean arterial pressure (MAP), systolic blood pressure (SBP), smoking status, CRF, drinking behavior, DM status, and weight status as potential predictors.

**Results:**

In total, 507 subjects had a stroke during an average follow-up period of 17 years (range=1–34 years). Logistic regression models showed that MAP values (*P*=0.043) in those aged 19–39 years; SBP (*P*<0.001), CRF (*P*=0.001), weight status (*P*=0.005), and alcohol consumption (*P*=0.001) in those 40–60 years old; and CRF (*P*=0.002), weight status (*P*=0.005), and DM status (*P*=0.037) in those over 60 years old were predictors of stroke.

**Conclusion:**

These findings suggest that, among individuals without a baseline HTN diagnosis, classic modifiable risk factors for stroke change across different stages of life.

## Introduction

The incidence of both incident and recurrent strokes is decreasing in the United States (US) and most industrialized countries,^1^,^2^ while recent data show that the risk of stroke-related mortality is leveling off, even decreasing, in the general population, but increasing in certain racial-ethnic groups and regions of the US.^3^ Notwithstanding, stroke still constitutes the fifth leading cause of death in the US,^4^ and one of the three major causes of mortality in industrialized countries.^5^ Age has been consistently reported as the leading risk factor for stroke, but many strokes remain susceptible to preventive measures since major risk factors, such as hypertension (HTN), diabetes mellitus (DM), tobacco smoking, excessive consumption of alcohol, obesity, and physical inactivity, are modifiable.^6^,^7^

Indeed, HTN has been described as the most important modifiable risk factor for cardiovascular disease (CVD).^8^ Blood pressure (BP) is defined by its pulsatile and steady components. The former, which can be assessed by pulse pressure (PP), represents the BP variation and is affected by early-pulse wave reduction, left ventricular (LV) ejection fraction, pulse rate, and large-artery stiffness.^9^,^10^ Meanwhile, the steady component is estimated by the mean arterial pressure (MAP) and represents the pulse rate, LV contractility, and vascular resistance and elasticity averaged over time.^11^ Elevated levels of systolic BP (SBP), MAP, and PP are associated with an increased risk of vascular diseases, such as stroke, coronary heart disease, congestive heart failure, peripheral vascular disease, and kidney disease.^12^,^13^ However, BP variability has been identified as a risk factor for stroke, independent of BP level.^14^,^15^

Other modifiable risk factors should also be considered to estimate the risk of stroke, but the interaction among them makes it difficult to elucidate their specific contribution to stroke events. First, the prevalence of HTN is elevated among subjects with high body mass index (BMI) and low cardio-respiratory fitness (CRF).^16^,^17^ Second, low levels of CRF have been consistently associated with low physical activity (PA).^18^,^19^ Although caloric intake contributes to obesity,^20^ decreases in leisure and occupational PA time are the main drivers of the significant increase in BMI during the last five decades.^21^ Finally, the contribution of other stroke risk factors (eg, DM, smoking status, and alcohol consumption) to the worldwide burden of stroke has been described.^22^

Because HTN, low CRF, obesity, DM, and unhealthy lifestyles are among the most important predictors of stroke risk, finding a common driver of these risk factors should be a priority in order to define preventive strategies of stroke.^23^ Several studies have assessed the importance of traditional risk factors in the prediction of stroke,^24^–^26^ but none have concurrently addressed the stroke-predicting ability of these risk factors across the lifespan in people without HTN. Thus, this study aimed to assess the importance of BP-related risk indicators, CRF, BMI, DM, and lifestyle factors as predictors of stroke in different stages of life.

## Materials and methods

### Study design and population

This was a prospective study including 33,254 men and 10,598 women from the Aerobics Center Longitudinal Study (ACLS) who did not have a diagnosis of HTN (self-reported physician diagnosed HTN at baseline). The participants were patients who visited the Cooper Clinic (Dallas, TX) in 1971–2004. At the beginning of the study, all participants were free of known myocardial infarction or stroke, and their resting electrocardiograms were normal; additionally, all participants were able to perform a stress test to at least 85% of their predicted maximum heart rate. To take part in the study, participants were required to answer at least one health survey by mail during follow-up. The majority of participants were Caucasian and of medium or high socioeconomic status. Additionally, participants’ written consent was needed to participate in the follow-up study. The study protocol was reviewed and approved annually by the Cooper Institute Institutional Review Board, and all study participants gave written informed consent. All aspects of the Strengthening the Reporting of Observational studies in Epidemiology (STROBE) guidelines were followed, and the paper was written accordingly.^27^

### Lifestyle factors and anthropometric and laboratory measurements

The medical examination and clinical measurements have been described elsewhere.^28^,^29^ Height and weight were measured on a standard physician’s scale, and BMI was calculated as weight (kg)/height (m)^2^. Participants were classified as underweight, normal weight, overweight, or obese according to the BMI cut-offs proposed by the World Health Organization (WHO).^30^ After a brief session of quiet rest, BP was measured by an auscultatory method with a mercury sphygmomanometer, diastolic BP (DBP) being recorded as the pressure at which the fifth Korotkoff sound appears.^31^ In addition to SBP and DBP, both PP (SBP-DBP) and MAP (SBP+1/3 PP) were determined; SBP, PP, and MAP were categorized by quartiles. For SBP, less than 110.00 constituted Q1, 110.00–117.99 was Q2, 118.00–125.99 was Q3, and more than 125.99 was Q4; for PP, less than 32.00 was Q1, 32.00–37.99 was Q2, 38.00–44.99 was Q3, and more than 44.99 was Q4; and, for MAP, less than 85.20 was Q1, 85.20–91.19 was Q2, 91.20–97.19 was Q3, and more than 97.19 was Q4. Information about smoking habits (current smokers or those who had quit smoking in the past 2 years were classified as smokers, and those who had never smoked or had quit smoking more than 2 years before the examination were classified as non-smokers) and alcohol consumption (heavy drinking defined as >14 alcoholic drinks per week for men and >7 alcoholic drinks per week for women) was collected. DM status was determined by self-reported DM or insulin use at baseline.

CRF was quantified as the duration of a maximal treadmill exercise test limited by symptoms using a modified Balke protocol.^32^ The treadmill speed was initially set at 88 m/min. The grade was 0% for the first minute and 2% for the second minute, then increased 1% each minute until 25 minutes. After 25 minutes, the grade did not change, and the speed increased 5.4 m/min each minute until test termination. Patients were encouraged to give maximal effort. CRF was classified as low or high according to the median age- and sex-specific distribution of the treadmill exercise duration.^33^,^34^

### Assessment of outcomes

We considered total stroke (combining fatal and non-fatal stroke) as the primary outcome. Following the International Classification of Diseases, we identified stroke using the Ninth Revision codes 430–434 and 436–438 for deaths that occurred before 1999 and using the Tenth Revision codes I60–I69 for deaths that occurred from 1999 to 2003. Health surveys to estimate the incidence of non-fatal stroke were administered six times between 1982 and 2004 (1982, 1986, 1990, 1995, 1999, and 2004), showing a 70% cumulative survey response rate across all periods of the ACLS. After the deceased were excluded from analysis, initial health status and clinical measures were similar between respondents and non-respondents, and between early and late interviewees. Total mortality rates were also similar between respondents and non-respondents. Although we could not perform a complete response-bias analysis, our observations revealed that respondents and non-respondents were generally similar.

Cases of stroke that were diagnosed by a physician were recognized using a case-finding question, asking the participants whether a physician had ever told them they had had a stroke. For positive answers, the year of diagnosis was collected. For participants who reported multiple strokes, we used only the first reported event for the analyses. This method of case finding has been previously used in longitudinal reports.^35^ The method shows 89% agreement between reported strokes and medical records.^36^

### Statistical analyses

Continuous variables are stated as the mean±SD, and categorical variables are stated as the number and percentage across the three age groups. Person-time for each participant was computed for each participant from the date of the baseline examination to the date of death; the date of a reported stroke; or December 31, 2003. Incidence rates of stroke were calculated as the number of cases divided by the person-time of follow-up by sex. Because the stroke risk of this cohort was low (less than 5%), logistic regression models^37^,^38^ were used with forward selection procedures for each age-specific group (18–39 years, 39–60 years, and ≥60 years), including the following variables as predictors: sex, PP, MAP, SBP, smoking status (yes/no), CRF by gender and age (metabolic equivalents: low/high), heavy drinking status (yes/no), DM status (yes/no), and weight status (normal weight/overweight/obese). Age-specific groups were determined based on differences in stroke prevalence according to NHANES 2011–2014.^8^ All analyses were conducted using Stata/SE software, version 15 (StataCorp, College Station, TX, USA). Statistical significance was defined as a *P*-value less than 0.05.

## Results

Table 1 presents the baseline characteristics of the study cohort for the overall population and across age categories. A total of 43,825 non-HTN individuals aged 18–100 years at baseline were followed-up for an average of 17 years (range=1– 34 years), and 507 cases of stroke (including 380 cases of non-fatal stroke) were registered. The incidence of stroke increased across age categories (0.35%, 95% CI=0.28–0.45, for 18–39 years; 1.42%, 95% CI=1.27–1.59, for 40–60 years; and 5.15%, 95% CI=4.28–6.13, for ≥60 years). Additionally, the prevalence of DM increased across age categories (1.50%, 95% CI=1.33–1.69, for 18–39 years; 2.45%, 95% CI=2.25–2.66, for 40–60 years; and 3.97%, 95% CI=3.21– 4.85, for ≥60 years). Similarly, mean levels of PP, MAP, and SBP progressively increased over increasing age categories. Finally, the overall prevalence of overweight/obesity was 58.49% (95% CI=57.95–59.02) in men and 22.04% (95% CI=21.26–22.84) in women, and this prevalence was higher in the 40–60-year-old group (54.00%; 95% CI=57.95–59.02) than in the other age groups.

The incidence density of stroke in events per 10,000 person-years was higher in men (7.43, 95% CI=7.35–7.51) than in women (6.15, 95% CI=6.01–6.28) in the overall sample (*P*<0.001) and progressively increased in every age group: 1.96 vs 1.71 for 18–39 years (*P*<0.001), 9.41 vs 7.39 for 40–60 years (*P*<0.001), and 37.49 vs 36.16 for ≥60 years (*P*=0.004).

Table 2 shows the long-term predictors of stroke (RR, 95% CI) from binary logistic regression models by age categories in the overall sample, in men specifically, and in women specifically. For the 18–39-year-old group, MAP was the only predictor of stroke (*P*_trend_=0.018). In this group, those in the third or fourth quartile of MAP had more than twice the stroke risk of those in the first quartile. For the 40–60-year-old group, SBP (*P*_trend_<0.001), CRF (*P*_trend_=0.001), weight status (*P*_trend_=0.005), and alcohol consumption (*P*_trend_=0.001) were included in the model. In this group, subjects in the third and fourth quartiles of SBP had a 2-fold and 3-fold increase in stroke risk, respectively, compared to those in the first quartile. Conversely, overweight and obese subjects had a 23% and 66% lower risk of stroke, respectively, than subjects with normal weight. Finally, for the ≥60-year-old group, low CRF (RR=1.83; 95% CI=1.25–2.70) and DM (RR=2.16; 95% CI=1.05–4.45) were predictors of stroke, while overweight (RR=0.64; 95% CI=0.43–0.96) and obesity (RR=0.29; 95% CI=0.12–0.68) decreased the risk of stroke.

## Discussion

This study provides evidence that the influence of long-term risk factors on future stroke is different throughout an individual’s lifetime. Thus, in logistic regression of stroke occurrence that include MAP, PP, SBP, weight status, lifestyle variables (smoking and drinking), DM, and CRF as predictors, the MAP value was the only parameter that increased the risk of stroke for those aged 18–40; SBP, low CRF, and heavy drinking status increased the risk of stroke for those 40–60 years old, while overweight and obesity decreased the risk; and low CRF and DM status increased the risk of stroke for those older than 60 years, while overweight and obesity decreased the risk.

Although numerous modifiable factors associated with stroke occurrence have been reported,^39^ no study has examined the predictive power of these factors at different stages of life. Recognizing that age is not a modifiable factor, the findings of this study reinforce the idea that the risk factors for stroke change throughout life and suggest that the algorithms to calculate the risk of stroke should be adapted depending on the stage of life.

The relationship between SBP and stroke has been widely described.^40^ Recent research has shown a *J-shaped curve* of the effect of SBP on the risk of stroke, demonstrating that both those with high levels and those with low levels of SBP are at high risk.^41^–^43^ Furthermore, in the last two decades, new BP markers, such as PP and MAP, have been proposed as potential risk factors for stroke^44^ that provide information on the relationship between SBP and DBP.^45^ Our data reinforce the conclusions of previous studies highlighting that stroke prevention before 60 years of age should be focused on SBP^40^,^46^ thus emphasizing the importance of MAP across the lifespan.^44^

The primary finding of our study is that, among elderly people free of HTN at baseline, overweight/obesity, high CRF levels, and non-diabetic status are the most important protective factors against stroke. Our results support previous studies in which the associations of CRF,^35^ obesity (the paradox of obesity),^18^ and DM with CVD and mortality have been independently reported.^18^,^35^,^45^,^46^ High CRF, obesity, and lack of DM could be framed in the definition of metabolically healthy obesity (MHO).^47^ Although the mechanisms underlying this metabolic profile are not yet clear, it has been suggested that the number of years that an individual has been obese, the person’s metabolic activity, and the distribution and histological characteristics of adipose tissue play a more important role than the amount of adipose tissue in determining metabolic health among obese individuals.^48^,^49^ Moreover, studies have reported that individuals with MHO have lower levels of C-reactive protein,^50^ higher levels of insulin,^51^ better CRF levels, and better general physical fitness than obese individuals with metabolic syndrome.^52^ Finally, scientific evidence supports the idea that individuals with MHO have less sedentary behavior time and higher levels of CRF than individuals with metabolically unhealthy obesity.^53^ These healthier lifestyle factors could partially explain the healthy metabolic profile of individuals with MHO. These findings reinforce previous research suggesting that individuals with MHO who have high levels of CRF are not at increased risk of ischemic stroke.^54^

This study has some limitations that must be emphasized: i) Some potential residual confounding could exist, as it was not possible to adjust for objectively measured PA, diet, or other possible confounding variables, such as intensity or duration of smoking, drug use or menopausal status, although it is doubtful that the residual confounding explains the entire association observed between the predictors used in this study and stroke; ii) the potential predictors of stroke were collected at the beginning of the study under the assumption that they are long-term predictors. Although it is likely that more active subjects with better CRF at the beginning of the study have healthier behavior throughout their lives,^55^ changes in these predictors might occur during the long follow-up period; iii) even if the subjects included in this analysis were classified as normotensive, the possibility of HTN under-diagnosis should be considered; iv) analyses based on the sub-type of stroke could not be performed due to the relatively small number of strokes reported in each sub-type; thus, the association between the variables included in this study and ischemic or hemorrhagic stroke could not be examined. Although separate models predicting cerebral hemorrhagic and ischemic stroke are currently advocated, it is not clear whether predictors differ among the subtypes.^56^ Future research should address the specificity of associations between potential predictors, such as PP, MAP, SBP, CRF, and weight status, and stroke subtypes; v) we had insufficient resources to verify all reported stroke events due to the cohort sample size and the wide geographic distribution; however, the agreement level between the participants’ self-reported history and their medical records seems to be acceptable (89%); and vi) the characteristics of the sample (mostly white women and men of medium and high socioeconomic status) may limit the generalization of our findings.

In summary, this study found evidence that MAP and SBP are associated with the incidence of stroke in young and middle adulthood in subjects without HTN. Modifiable risk factors such as CRF, weight status, and alcohol consumption emerge as predictors in middle adulthood. Likewise, it is worth highlighting the main finding of this study: among those over 60 years old, weight status, CRF, and DM are the best predictors of stroke. Knowledge and awareness of stroke risk factors throughout life is of great importance from a clinical point of view because this can help promote prevention efforts by potentially emphasizing lifestyle-directed recommendations according to the age of the patient. Nonetheless, although the benefits of weight-loss interventions among overweight and obese individuals could be expanded beyond cardiovascular events, our results indicate that interventions aimed at preventing cardiometabolic risk factors among older individuals should be implemented regardless of weight status, particularly among people over 40 years old. Moreover, future lines of research should clarify which health interventions could be the most effective in each life stage to prevent stroke incidence. It is also necessary to elucidate the role of overweight and obesity as predictors of stroke through randomized controlled trials aimed at assessing the effects of weight reduction on the risk of stroke, particularly in obese patients.

## Figures and Tables

**Table 1:** Baseline characteristics of the study participants

	Overall (N=43,852)	18–39 years (N=18,911)	40–59 years (N=22,648)	≥60 years (N=2,293)
Age (years)	42.84±9.87	33.98±5.00	48.04±5.06	64.40±4.96
Men	33,254 (77.20)	14,188 (75.03)	17,350 (76.61)	1,716 (74.84)
BMI	25.43±3.88	25.00±3.98	25.76±3.79	25.61±3.53
CRF (METs)	11.22±2.60	11.93±2.58	10.84±2.46	9.13±2.26
SBP (mmHg)	117.80±13.31	115.55±12.25	118.85±13.45	125.98±15.52
DBP (mmHg)	78.69±9.34	76.91±9.10	80.01±9.31	80.30±9.18
PP (mmHg)	39.11±10.24	38.64±9.79	38.84±10.09	45.69±12.81
MAP (mmHg)	91.60±9.68	89.66±9.16	92.83±9.76	95.38±9.98
Cases of stroke	507 (1.16)	67 (0.35)	322 (1.42)	118 (5.15)
DM status	930 (2.12)	284 (1.50)	555 (2.45)	91 (3.97)
Current smoker	7,032 (16.04)	3,258 (17.23)	3,580 (15.81)	194 (8.46)
Heavy drinker	5,625 (12.83)	2,649 (14.01)	2,787 (12.31)	189 (8.24)
Weight status				
Normal weight	22,067 (50.32)	10,556 (55.82)	10,418 (46.00)	1,093 (47.67)
Overweight	17,058 (38,90)	6,569 (34.74)	9,528 (42.07)	961 (41.91)
Obesity	4,727 (10.78)	1,786 (9.44)	2,702 (11.93)	239 (10.23)
PP quartiles				
<32.00 mmHg	12,160 (27.73)	5,387 (28.49)	6,432 (28.40)	341 (14.87)
32.00–37.99 mmHg	15,227 (34.72)	6,795 (35.93)	7,884 (34.81)	548 (23.90)
38.00–44.99 mmHg	7,684 (17.52)	3,290 (17.40)	3,968 (17.52)	426 (18.58)
≥45.00 mmHg	8,672 (19.78)	3,391 (17.93)	4,309 (19.03)	972 (42.39)
MAP quartiles				
<85.20 mmHg	12,295 (28.04)	6,540 (34.58)	5,371 (23.72)	384 (16.75)
85.20–91.19 mmHg	12,314 (28.08)	5,578 (29.50)	6,186 (27.31)	550 (23.99)
91.20–97.19 mmHg	10,615 (24.21)	4,114 (21.75)	5,872 (25.93)	629 (27.43)
≥97.20 mmHg	8,504 (19.39)	2,616 (13.83)	5,164 (22.80)	724 (31.57)
SBP quartiles				
<110.00 mmHg	9,921 (22.62)	5,049 (26.70)	4,638 (20.48)	234 (10.20)
110.00–117.99 mmHg	11,350 (25.88)	5,285 (27.95)	5,660 (24.99)	405 (17.66)
118.00–125.99 mmHg	11,499 (26.22)	4,902 (25.92)	6,044 (26.69)	553 (24.12)
≥126.00 mmHg	10,976 (25.03)	3,630 (19.20)	6,251 (27.60)	1,095 (47.95)
Low CRF status	20,977 (47.84)	9,154 (49.99)	10,739 (47.42)	1,084 (47.27)

**Note:** Mean±SD for continuous variables and number (%) for categorical variables.

**Abbreviations:** BMI, body mass index; CRF, cardiorespiratory fitness; DBP, diastolic blood pressure; DM, diabetes mellitus; MAP, mean arterial pressure; METs, metabolic equivalents; PP, pulse pressure; SBP, systolic blood pressure.

**Table 2:** Long-term predictors of stroke in logistic regression models^a^ including the 43,854 participants from the ACLS without a diagnosis of HTN at baseline

	Cases/n	Forward selection	RR (95% CI)	*P*-value
18–39 years	67/18,911	MAP		0.018
		Q2	1.17 (0.57–2.40)	
		Q3	2.23 (1.15–4.33)^b^	
		Q4	2.51 (1.23–5.14)^b^	
40–60 years	322/22,648	SBP		<0.001
		Q2	1.57 (1.00–2.45)	
		Q3	2.83 (1.88–4.27)^b^	
		Q4	3.60 (2.40–5.39)^b^	
		Low CRF	1.50 (1.19–1.89)^b^	0.001
		Weight status		0.005
		Overweight	0.77 (0.61–0.98)^b^	
		Obese	0.54 (0.36–0.80)^b^	
		Heavy drinking status	1.59 (1.20–2.11)^b^	0.001
≥60 years	118/2,293	Low CRF	1.83 (1.25–2.70)^b^	0.002
		Weight status		0.005
		Overweight	0.64 (0.43–0.96)^b^	
		Obese	0.29 (0.12–0.68)^b^	
		DM status	2.16 (1.05–4.45)^b^	0.037

**Notes:**

a Forward selection variables.

b Statistically significant coefficients (*P*<0.05). The following variables were included as candidates in the model in each category of age: sex, MAP, PP, SBP, smoking status (yes/no), age and gender-adjusted CRF (low/high), heavy drinking status (yes/no) DM status (yes/no), and weight status (normal weight/overweight/obese).

**Abbreviations:** ACLS, Aerobics Center Longitudinal Study; CRF, cardiorespiratory fitness; DM, diabetes mellitus; HTN, hypertension; MAP, mean arterial pressure; PP, pulse pressure; SBP, systolic blood pressure.

## Abbreviations

ACLS: Aerobics Center Longitudinal Study
BMI: body mass index
BP: blood pressure
CRF: cardiorespiratory fitness
CVD: cardiovascular disease
DM: diabetes mellitus
HTN: hypertension
LV: left ventricular
MAP: mean arterial pressure
MHO: metabolic healthy obesity
PA: physical activity
PP: pulse pressure
SBP: systolic blood pressure
WHO: World Health Organization
